# Bibliographical Department

**Published:** 1880-06

**Authors:** 


### BIBLIOGRAPHICAL DEPARTMENT.

"Nothing extenuate, nor set down aught in malice."

"A Practical Treatise on Sea-Sickness: Its Symptoms, Nature and Treatment. By George M. Beard, A. M., M. D., Fellow of the New York Academy of Medicine, of the New York Academy of Sciences, Vice-President of the American Academy of Medicine, Member of the American Neurological Association, of the American Medical Association, of the New York Neurological Society, Author of "Nervous Exhaustion," (Neurasthenia), "Our Home Physician," etc. New York: E. B. Treat, No. 757 Broadway, 1880. All those who go down to the sea in ships, should be sure to supply themselves with this well written and valuable volume, and just about this season of the year, their name is legion, who, intent upon pleasure, relaxation or business, are preparing for sea voyages of various duration, from several hours to as many days; but whether long or short, almost all will be called upon to pay tribute to old Neptune, and will respond to the summons, however reluctantly, unless they have profited by the excellent rules and suggestions for treatment contained in Dr. Beard's admirable little book.

"Investigation and Experience of M. Shawtinbach, at Saar Soong, Sumatra. A. Ret. or Sequel to the Manatillans. San Francisco: Joseph Winterburn & Company, Printers and Electrotypers, 417 Clay street, between Sansom and Battery, 1879." We are indebted to Dr. E. R. Smilie, for a copy of the foregoing book. Time has not allowed of its perusal to any extent, but reference has been made to a few of the pages designated as answering certain questions asked of the "The President and Laymen of the S. F. A. S., by the author, and they are found "O. K." Disciples of the Darwinian hypothesis, and pupils of the theory of evolution, would probably find much to interest *them* in its pages, as almost any of them could discover the rudiments of a tail, and often a whole *tale* in every chapter referring to thier *common ancestors.* Caudal appendages seem to have a *waggish* tendency.

The Problem of Insanity: A paper read before the New York Medico-Legal Society, March 3d, 1880, by George M. Beard, A. M., M. D., Member of the New York Medico-Legal Society; Fellow of the New York Academy of Medicine; Vice President of the American Academy of Medicine; Member of the Neurological Association, etc., etc. This paper, like everything emanating from the pen of Dr. Beard, is worthy of careful perusal and attentive consideration, and is therefore adapted to all classes of the profession before which it was read.

We are in receipt of the printed "Report of the Proceedings for establishing a Board of Commissioners in Lunacy for the State of New York," and respectfully commend it to the proper authorities in Maryland, as well worthy of their imitation and adoption also.

A Lecture on Median Lithotomy, delivered before the Brooklyn Anatomical and Surgical Society, by James L. Little, M. D., N. Y., Professor of Surgery in the University of Vermont and New York City, is a well written and interesting pamphlet.

We have received from the publishers a copy of the *Index Medicus*, and a request that we should aid the enterprise as much as we could.

We do not hesitate to commend the publication, and in fact as many of our readers as may be in circumstances to do so, to subscribe to the work as promptly as practicable. It is edited with much ability by Dr. John S. Billings, Surgeon, U. S. Army, and Dr. Robert Fletcher, M. R. C. S. Eng., and published at 3 dollars per annum, by F. Leypoldt, New York City.

We welcome the "Pacific Medical and Surgical Journal, Drs. Henry Gibbons, Sr. and Jr., editors and proprietors. Published monthly in San Francisco, Cal., at 3 dollars a year in advance." This is an old, orthodox, and well edited journal.

"The Sanitary Journal" of Toronto, Canada, has been received and placed on the list of exchanges, also. It is edited by Edward Playler, M. D., and published monthly. Address Sanitary Journal, Toronto.

Vol. 1, No. 5, of the "Dental Jairus" a monthly journal edited by W. O. Thrailkill, D. D. S., of Sacramento, Cal., who also carries on a dental depot. We are happy to see our former student in such a neat dress, with the appearance of prosperity.

Received a copy of the "Transactions of the American Dental Association, at the Ninetieth Annual Session, held at Niagara Falls, August 5th, 6th, 7th and 8th, 1879." Its 134 pages are handsomely printed and bound in paper cover. Many thanks to Dr. Geo. H. Cushing, of Chicago, chairman of the publication committee, to whom we are kindly indebted.

"The Dentist: a Popular Treatise on the Care of the Teeth." By O. H. Jarvis, M. D. S., of N. Y. City, second edition. It is written in an entertaining style, illustrated, bound in cloth, and highly commendable to the laity.

